# Supplementary material for: A neural network approach to predict opioid misuse among previously hospitalized patients using electronic health records
Source: PLoS One. 2024 Aug 28;19(8):e0309424. doi: 10.1371/journal.pone.0309424 (PMC11356447; doi:10.1371/journal.pone.0309424)
Supplement: Attachment — Submitted filename: Review Comments PONE-D-24-07080.pdf [file pone.0309424.s001.pdf]

Dear Authors,

After going through your paper "A Neural Network Approach to Predict Opioid Misuse among Previously Hospitalized Patients using Electronic Health Records" I can see that it has high value and address a major issue i.e., opioid misuse. The paper is unique and presents how electronic health records (EHR) could be used to predict opioid misuse using neural network approach. I agree that opioid misuse is a significant problem and can lead to cases of opioid overdose and deaths. Therefore, if earlier identification of opioid misuse could be done among previously hospitalized patients it could further benefit them from getting risk of opioid overdose.

After going through your paper, I found most of the contents within the paper is well explained and I genuinely commend authors for that. However, I also found that the authors didn't follow the author guidelines (https://journals.plos.org/plosone/s/submission-guidelines) properly before submitting the paper. Because of the content's irregularity, the paper can get confusing and difficult to read. I recommend authors to strictly follow such guidelines so that it maintains clarity and readability among researcher and even readers. As of current, it looks like a thesis dissertation file. Similarly, the authors also haven't included significant components such as "the introduction of neural network, its approach, EHR in the introduction section." This creates confusion and shows lack of coherence. I recommend authors to fully revise the paper following the guidelines. I have few comments for the authors. These comments are simple as it mainly focuses with the adhering journal guidelines along with clarity and readability of paper.

Comments:

Introduction

1. Line 11-12: "In the United States, nearly 10 million individuals misuse opioids annually National Center for Drug Abuse Statistics (2021)."
   This line creates confusion on whether it's an entire sentence or the later one is intext-citation. Please amend so that clarity can be maintained. Please maintain references thoroughly. The author guidelines mention on using "Vancouver" (See under style and format), however you seem to have used author-date style.

2. Line 12-13: "Opioid misuse occurs when an individual 1) takes opioids in different/larger quantities than prescribed by their physician, or 2) takes opioids without a prescription."
   Please cite this sentence too as this sentence provide useful information.

3. Line 35-39: With respect to your topic, I don't find any introduction on the idea of neural network, its approach, its meaning and purpose. Similarly, I don't see any introduction to EHR and its purpose in this sense. The authors could have also provided a little context to machine learning models and their importance in predicting such misuse.

4. Line 41-45: "The rest of this paper is structured as follows. Section 2 is the Literature Review and explores opioid risk assessment tools and machine learning. Section 3 contains our Research Questions. Section 4 is the Experimental Design and we present the OMPS System.

Section 5 details the Experimental Results and Discussion. Section 6 delivers our Conclusions, Limitations and Future Directions. Finally, Sections 7 and 8 contain the Abbreviations and Declaration statements respectively”.

This is not needed. We don’t mention such things in introduction section. Better omit this.

5. Section 2 and its subsection is not required. Please follow the author guidelines properly. Within this section, you have mentioned all the overall variables/checklist (taxonomy of opioid risk assessment tools) and machine models. This perfectly sets up for the upcoming section. But a specific literature review section is not needed, please amend this portion within your introduction, methodology sections. Including this in your earlier and later sections, make your paper must robust and readable.

6. Section 2.2 Line 132-135 explains on the machine learning and Line 137-139 shows the meaning of EHR and its use. You can include this in your introduction section.

7. Section 2.3 “Research gaps” ideas could have been incorporated within the introduction section.

8. Section 3 “Research questions” could also have been incorporated within the introduction section.

9. Section 4 should be methodology not experimental design and only the authors should highlight it as an experiment design study.

10. Section 5: Please rename it to as “Result and discussion”. I acknowledge the authors idea of presenting the results as per the research questions.

11. The limitations, further implication sections are well presented in accordance with study results and objectives.

12. Please look out for potential grammatical errors, abbreviation and spelling mistakes.

I commend authors for providing such detailed explanation on the topic. However, your work needs further revision to improve its clarity and readability.
