# Supplementary material for: A neural network approach to predict opioid misuse among previously hospitalized patients using electronic health records
Source: PLoS One. 2024 Aug 28;19(8):e0309424. doi: 10.1371/journal.pone.0309424 (PMC11356447; doi:10.1371/journal.pone.0309424)
Supplement: Attachment — Submitted filename: Comments.docx [file pone.0309424.s002.docx]

Comments

1)Overall, the writing needs to be improved in terms of language and grammar.

2)I feel many unnecessary sections could be trimmed and included in the introduction and methodology. The manuscript is very lengthy and should be reduced to a readable pace.

3) Also, please check on the referencing system.

4)Line 11- The line seems to be incomplete. Please make sure it is clear.

5)Line 14- Opiate use disorder- Probably you could provide a clear description on this and what are the consequences.

6) Line 32- What is the defination of universal screening that you meant? Or is it based on a standardised protocol?

7) In Line 32, you mentioned that there is a lack of resources to administer questionnaires but under Figure 1 (Opiate risk assessment tools), there is questionnaire. So which one you are referring to? Please be clear.

8) Line 441- “This finding also aligns with previous research.”

Where are the references?

9) Please adhere to the manuscript formatting for the article. It looks more like a mini-thesis to me.
